# Interspecific Interactions and the Scope for Parent-Offspring Conflict: High Mite Density Temporarily Changes the Trade-Off between Offspring Size and Number in the Burying Beetle, *Nicrophorus vespilloides*

**DOI:** 10.1371/journal.pone.0150969

**Published:** 2016-03-17

**Authors:** Ornela De Gasperin, Rebecca M. Kilner

**Affiliations:** Department of Zoology, University of Cambridge, Cambridge, CB2 3EJ. United Kingdom; Leiden University, NETHERLANDS

## Abstract

Parents have a limited amount of resources to invest in reproduction and commonly trade-off how much they invest in offspring size (or quality) versus brood size. A negative relationship between offspring size and number has been shown in numerous taxa and it underpins evolutionary conflicts of interest between parents and their young. For example, previous work on vertebrates shows that selection favours mothers that produce more offspring, at the expense of individual offspring size, yet favours offspring that have relatively few siblings and therefore attain a greater size at independence. Here we analyse how this trade-off is temporarily affected by stochastic variation in the intensity of interspecific interactions. We examined the effect of the mite *Poecilochirus carabi* on the relationship between offspring size and number in the burying beetle, *Nicrophorus vespilloides*. We manipulated the initial number of mites in the reproductive event (by introducing either no mites, 4 mites, 10 mites, or 16 mites), and assessed the effect on the brood. We found a similar trade-off between offspring size and number in all treatments, except in the '16 mite' treatment where the correlation between offspring number and size flattened considerably. This effect arose because larvae in small broods failed to attain a high mass by dispersal. Our results show that variation in the intensity of interspecific interactions can temporarily change the strength of the trade-off between offspring size and number. In this study, high densities of mites prevented individual offspring from attaining their optimal weight, thus potentially temporarily biasing the outcome of parent-offspring conflict in favour of parents.

## Introduction

**Fig 1:**
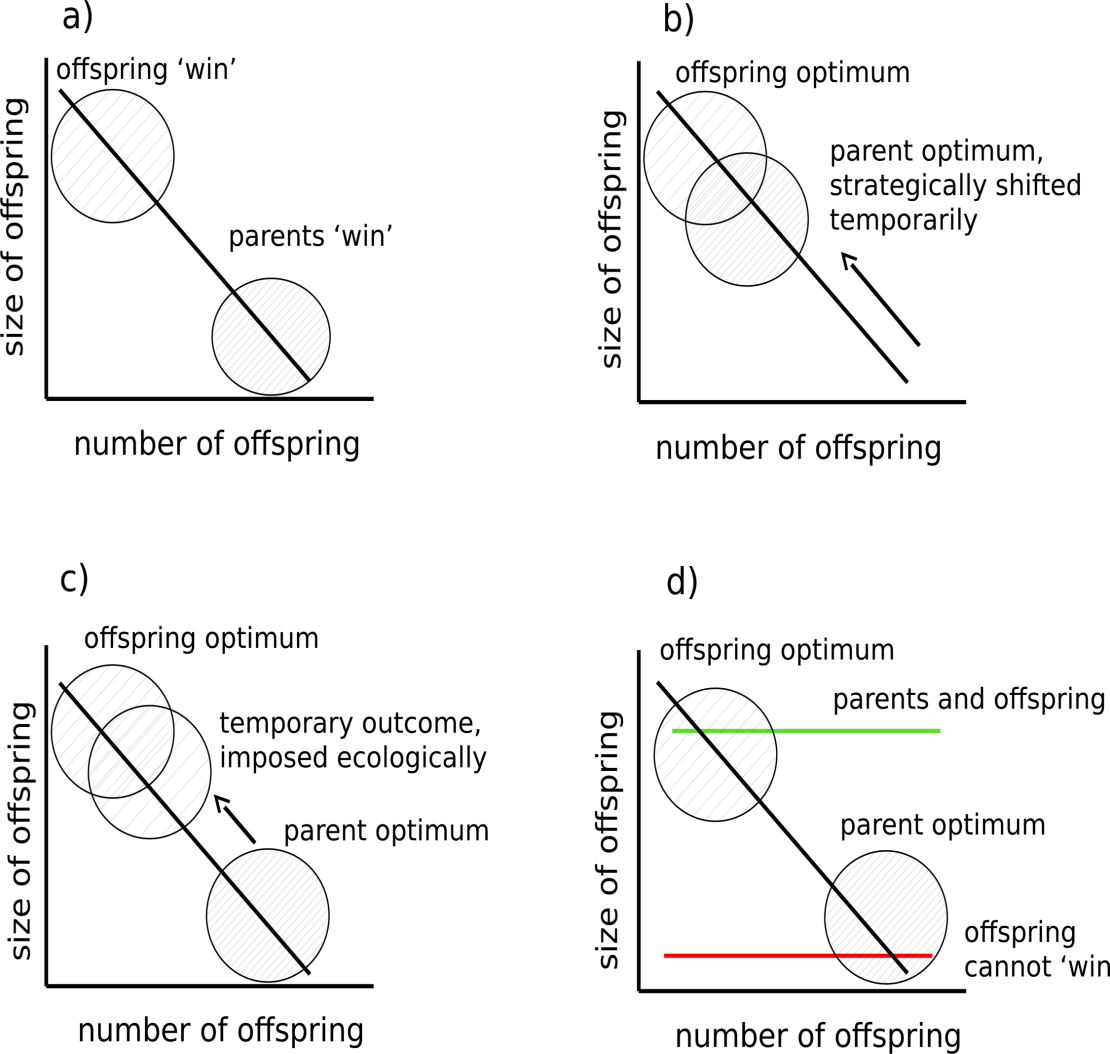
Parent-offspring conflict over the trade-off between offspring size and number. a) Empirical analyses reveal that selection can act differently on genes in parents and offspring in the longer term to favour a different optimal balance between offspring size and brood size. Optimal outcomes for each party are labelled: offspring ‘win’ and parents ‘win’. b) Fluctuating ecological conditions can temporarily favour one party by changing the positioning of the size-number trade-off. In some scenarios, it may be temporarily aligned closer to the offspring’s optimum (as illustrated here), in others it remains closer to the parent’s optimum. c) In some situations, ecological conditions might even impose an outcome that is closer to the offspring’s optimum and against the parent’s evolutionary interest. d) Alternatively, fluctuating ecological conditions might change the gradient of the trade-off. At one extreme (shown with the green line), caused by very high food abundance for example, it may remove any conflict over offspring size completely because the optima for parents and offspring are temporarily closely aligned. At the other extreme (shown with the red line), caused by sudden limited food availability for example, it may temporarily prevent offspring from ever attaining investment close to their optimum.

Parents have a limited amount of resources to invest in reproduction, and so must balance how many offspring they decide to produce against investment in their size (or quality) [1]. This generates a negative correlation between offspring size and number, evidence of which has now been found in a wide range of taxonomic groups, including mammals, reptiles, amphibians, birds, plants, insects, crustaceans, and humans (e.g. [2–4]). Detailed analyses of long-term datasets gathered from vertebrate populations have shown that the trade-off between offspring size and number can generate evolutionary conflicts of interest between parents and their young [5–7]. Parent-offspring conflict arises because selection acts differently on genes expressed in parents and in their young, favouring a different optimal trade-off between offspring size and brood size in each party (reviewed by [8]). In this context, and over the longer term, selection favours mothers that produce more offspring at the expense of individual offspring size, yet favours offspring that have relatively few siblings and therefore attain a greater size at independence ([5–7] Fig 1A).

At first sight it might seem that offspring are doomed to lose this particular conflict of interests because mothers surely have the greater influence over the size of the young they produce [7,9]. However, there are several ways in which fluctuating ecological conditions could temporarily tip the outcome of this evolutionary conflict in the offspring’s favour (see Fig 1). For example, a change in the wider environment might change the fitness costs and benefits associated with investment in offspring size, forcing a strategic adjustment by mothers in the trade-off they strike between offspring size and brood size [10]. This could align the evolutionary interests of parents and offspring in the shorter term [11], and temporarily shift investment towards the long-term offspring optimum (Fig 1B). A possible example of this comes from Soay sheep *Ovis aries*. Female sheep in better environmental conditions tend to produce twins, with each offspring attaining a relatively low mass whereas those in worse conditions produce a single, larger lamb [12]. Nevertheless, the long-term maternal optimum is for sheep to produce twins [6].

In some scenarios, it is even possible that ecological conditions enable offspring to ‘win’ this conflict, against the evolutionary interests of their mothers (Fig 1C). Suppose, for example that predators temporarily and selectively target smaller offspring. In this case offspring that temporarily achieve investment levels closer to their optimum would have an advantage.

In these two examples, the extent of variation in the wider environment does not contribute to the total amount of resources allocated to offspring and it simply causes variation in where individual families sit on the curve describing the trade-off between offspring size and number. But it is also possible that environmental conditions cause temporary fluctuations in the gradient and elevation of this curve (Fig 1D), changing the area underneath it in proportion to the total amount of resources available for producing offspring. At one extreme, if parents are temporarily swamped with resources, the gradient of the offspring size-number trade-off may flatten at a high intercept, potentially causing any parent-offspring conflict over offspring size to disappear altogether (Fig 1D) because offspring are able to achieve investment close to their optimum and brood size is also large (e.g. [4]). At the other extreme, in poor quality environments where resources are limited, the area under the curve will be correspondingly smaller [13]. This might change the slope or the elevation of the trade-off curve, or both. If resources are sufficiently scarce, the trade-off curve will flatten completely but this time at a very low intercept. Here offspring could never attain a size that is close to their optimum, though parents may still produce their optimal number of offspring (Fig 1D).

In this paper, we consider how the intensity of interspecific interactions might temporarily influence the trade-off between offspring size and brood size as a first step towards understanding how they could potentially bias the outcome of parent-offspring conflict in the burying beetle *Nicrophorus vespilloides*. We focus specifically on the interaction between the beetle and its phoretic mite, *Poecilochirus carabi*. There is no explicit evidence yet from burying beetles to show that selection on parents in this species favours the production of many, smaller offspring, and that selection on offspring favours the production of fewer, larger larvae. Nevertheless, such a scenario is not implausible.

Burying beetles require the carcass of a small vertebrate in order to breed, and there can be intense competition for this key breeding resource [14,15]. Disputes over carcass ownership are resolved through fights, which larger individuals (of either sex) typically win [15,16]. From an individual offspring’s point of view, therefore, selection will probably favour a larger body size because it is then almost guaranteed to acquire a carcass and reproduce. Nevertheless, it is unlikely that ownership of every single carcass will be disputed in nature [17]. Furthermore smaller individuals can gain some reproductive success through alternative mating strategies [18–20]. For these two reasons, a large body size is not essential for successful reproduction [21]. Selection on parents could therefore favour the production of more, slightly smaller offspring because this could feasibly yield more grand-offspring than investment in fewer, larger offspring.

The life cycle of the burying beetle is closely linked to that of its phoretic mite *P*. *carabi* [22–24]. Just like the burying beetle, these mites require carrion for reproduction, though unlike the burying beetle they lack the means to travel between breeding opportunities. Mite deutonymphs (the phoretic stage of the mite) attach themselves to the beetles, and the beetles transport them between breeding events. Once a beetle arrives at a carcass, the mites disembark, moult, mate, reproduce and die [23,24]. The mites apparently feed on the carrion and so potentially compete for this resource with the burying beetle, changing evolutionary interactions within the beetle family as a result [25,26]. The next generation of mites disperses mainly with the parents as they fly off at the end of the reproductive event [24] in search of another carcass [23].

Note that burying beetles parents lay more eggs than they can raise as larvae and then regulate brood size through partial filial cannibalism [27]. Parents thus have a large degree of control over the body-size brood size trade-off [27]. Nevertheless, mites can potentially change the balance of power by changing this trade-off. For example, in previous work, we added a fixed number of mites (ten) to a carcass when pairing beetles to breed [25]. Some mites reproduced prolifically alongside the beetle, others less so. Thus by the time parents dispersed away from the breeding attempt there were variable numbers of mite progeny present. We found that the trade-off between offspring size and number in the burying beetle was related to the number of progeny mites left at the end of the reproductive event [25]. A large number of mite progeny was associated with fewer, larger larvae (probably because the mites attacked larvae and directly reduced brood size) whereas with fewer mites, more larvae were produced but they were smaller [25]. These experimental data are therefore similar to hypothetical effects on the trade-off between brood size and offspring size shown in Fig 1C (though they are not an explicit test of this idea). At higher densities still, mites could deplete resources for larvae on the carcass to such an extent that the slope or elevation of this trade-off is changed as a result (as illustrated in Fig 1D).

In natural populations, the number of mites carried by each adult can vary widely ([23]; personal observation). The aim of the current study was to determine how variation in the density of mites present at the start of reproduction affects the strength of the trade-off between offspring size and number in the burying beetle. We predicted that this sort of stochastic ecological variation might be powerful enough to temporarily change the gradient of the trade-off between offspring size and number, as illustrated in Fig 1D, though we had no *a priori* predictions about the direction of any change in the elevation of the gradient.

## Methods

### Study species

#### Burying beetles

All the beetles used in these experiments came from a stock population founded in 2005. Every year new field beetles are brought into the colony between spring and autumn, and bred with our population colony to avoid inbreeding. We collected field beetles from Wicken Fen (52.3108° N, 0.2913° E, with permission from the National Trust, permit number 1166 and 1811), and from Byron's Pool (52.1790° N, 0.0950° E, with permission from the Cambridge City Council). Before introducing field beetles we removed any mites on them (see below), and thus kept our burying beetle colony separate from our mite colony. All beetles were kept in small plastic containers (12cm x 8cm x 2cm) filled with moist soil and fed twice a week with small pieces of minced beef. The colony was maintained in a lab at 20°C and on a 16:8 light to dark cycle. Adult beetles were bred when they were between two and three weeks old in plastic breeding boxes (17cm x 12cm x 6cm) filled two-thirds with moist soil and furnished with a mouse carcass. (Note that these general methods mean that all the beetles used in the experiments described here developed as larvae in an environment without mites).

Burying beetles exhibit biparental care. Together, the parents prepare and bury the carcass of a small vertebrate by ripping off any fur or feathers, smearing it with antibacterial exudates, and rolling it into a ball [14,15], thereby transforming it into an edible nest for their larvae [14,15]. The carcass provides a finite resource for nourishing offspring and helps generate the negative trade-off between larvae size and larvae number (see [28,29]).

#### Mites

Natural populations of burying beetles interact with several species of phoretic mites [23]. The *Poecilochirus carabi* species complex comprises several species that are morphologically similar [30,31]. We focused on the *Poecilochirus carabi* complex because they are the most common mites we find on naturally caught burying beetles at our field sites (most of the mites found on *N*. *vespilloides* beetles in nature are *P*. *carabi* sensu stricto [32]). These mites are readily apparent as the deutonymphs (the phoretic stage) are large, very mobile and aggregate on the beetle’s head and thorax. We harvested *P*. *carabi* deutonymphs from field-caught *N*. *vespilloides* by anaesthetising the burying beetle with CO_2_, and using a brush and tweezers to remove and count the mites. Since we harvested deutonymphs from field-caught *N*. *vespilloides*, the mites used in the experiment are representative of the naturally occurring *P*. *carabi* on our study species.

Once separated from the burying beetle, we kept the mites in plastic containers (17cm x 12cm x 6cm) filled with moist soil, and fed them once a week with minced beef. We kept the containers inside cupboards, with one burying beetle living alongside the mites in each plastic container (this beetle was not used in any experiments), as this facilitates mite collection for experimental purposes. We bred the mite colony once a month. For this, we placed spare pairs of burying beetles from our stock population to breed on the carcass of a mouse and introduced ~15 mites into the breeding box (17cm x 12cm x 6cm). At the end of the reproductive event we anaesthetised both beetles and kept the mites that were dispersing on them.

#### Experimental design

We placed pairs of sexually mature (between two and three weeks old), virgin beetles to breed inside a plastic container with 2 cm of soil in four different treatments: with zero, four, ten, or sixteen mites. Each wild-caught beetle of *N*. *vespilloides* carries on average 4–8 deutonymphs [24] so all of the mite densities used in this experiment are likely to occur commonly in natural breeding events when pairs of beetles breed together (i.e. each carrying between 4 and 8 deutonymphs). Furthermore, mite density at a carcass is likely correlated with the extent of competition among *Nicrophorus* beetles for a carcass. Each beetle that visits a carcass brings mites that remain on that carcass. So higher mite densities may reflect higher competition (De Gasperin, unpublished PhD thesis, 2015; P. Hopwood, personal communication). To introduce the mites we carefully placed each deutonymph on the soil surrounding the mouse at the same time as we introduced the mouse. We bought dead white mice from livefoodsdirect.co.uk.

Because parental size and condition can influence the trade-off between offspring number and size (e.g. [4,33]), we decided to control for parental condition by placing tetrads of brothers to breed with unrelated tetrads of sister distributed across the four treatment groups. For example, we paired brothers of family x with sisters of family y, and placed one pair of male ‘x’ and female ‘y’ in each treatment. We recorded this ‘pair code’ and included it as a random effect in our analysis (see below). We weighed the carcass at the start of the reproductive event, and again once carcass preparation was finished (56 h after pairing). The mass of the carcasses at the beginning of the experiment was between 8–15 g carcass (mean = 9.99 g; SD = 0.85). There was no significant difference among treatments in the mass of the carcass provided (χ^2^_3_ = 4.25; *p* = 0.23). On the afternoon before the larvae hatched we measured clutch size by counting all the eggs observable in the bottom of the breeding box (around 56 h after pairing). Previous observations of breeding events (without mites) in our lab have shown that this estimate of clutch size is highly correlated with the actual clutch size (Pearson’s correlation *r* = 0.908; M. Schrader unpublished data).

Males from *N*. *vespilloides* usually abandon the nest several days before the female does [26]. Furthermore, the presence of the mites accelerates male desertion, at least when 10 mites are added at the start of reproduction [26]. However, when we designed the experiment, we did not know how higher mite densities might affect the desertion time of the male. We did not want to remove males at a random, unnatural time, but nor did we want to remove males at a different time in each experimental treatment and thereby introduce a potential confounding effect. We therefore decided to solve this problem by following the common practice (e.g. [34,35]) of leaving both parents in the breeding box until the end of the breeding event.

At the end of the reproductive event (eight days after pairing) we opened all the breeding boxes and collected all the larvae from each box. We counted all the larvae and weighed the brood. We also counted any dead larvae found in the soil (these were only 3^rd^ instar larvae). We anaesthetised all the parents at this point with CO_2,_ and removed and counted all the deutonymphs dispersing on them using tweezers and a brush. We also measured the pronotum width of all parents at this point to assess their body size, but we did not keep the parents thereafter. We handled all the beetles with care. We performed this experiment in two blocks, with a total of 30 replicates per treatment.

#### Statistical analysis

Data from this experiment can be found in S1 Table. We had a total of 24 successful replicates in the 'without mite' treatment, 28 in the '4 mite' treatment, 28 in the '10 mite' treatment, and 27 in the '16 mite' treatment. We analysed all the data with the statistical program R [36], with general mixed effects models (lme4 package; [37]), using the 'lmer' function. In every model we included the ‘pair code’ as a random effect nested within the block. We reduced every model using the Akaike Information Criterion (AIC; [38]), and checked the distribution of the residuals from the final models. We obtained *p* values for the general effects using the ‘Anova’ function with type 'III' sum of squares from the ‘car' package [39], and for individual comparisons using the 'summary' function [37].

To determine whether the initial mite density influenced the trade off between brood size and offspring size, we used average larval mass as a response variable and looked explicitly for an interaction between the size of the brood and the mite density treatment. In addition, we controlled statistically for the mass of the carcass after preparation, and the size of the parents by including these measures as covariates. We also ran individual models within each 'mite treatment' to obtain estimates of the coefficients and *p* values between the size of the brood and the average larval mass for each 'mite treatment'. In these models we only included the size of the brood and the mass of the carcass after preparation as covariates, and the block as a random effect. We also analysed whether there was an interaction between the carcass mass (after preparation) and the different mite treatments on a) final brood mass, b) final brood size, and c) average larval mass. In the last model we also included the size of the brood as a covariate. In all of our analyses we only included data from the beetles that bred successfully.

To better understand how any change in the trade-off between brood size and offspring size may have arisen, we analysed variation in clutch size, brood size and the mass of the brood. In these analyses, our explanatory variables were the mite density treatment, the mass of the carcass after preparation, and the size of the parents. We also analysed whether the number of dead larvae found at the end of the reproductive event varied according to the initial mite density. For this, we ran a generalised linear mixed effects model with a Poisson distribution (using the glmer function [37]), and included as fixed effects the treatment, and the mass of the carcass.

Finally, we analysed whether variation in the final number of mites explained variation in the success of the brood, for comparison with our previous study see [25]. For these tests, we used as response variables the final size and mass of the brood, the clutch size, and the average larval mass. We included as explanatory variables the mass of the carcass after preparation and the final number of mites (variable obtained by adding the mites dispersing on the male and on the female beetle, and log transforming this variable). We did these analyses for all the mite treatments combined, and also individually for each mite treatment. When analysing the average larval mass we also included the size of the brood as a covariate. We included as a random effect the ‘pair code’ nested within the block when analysing data from several treatments together, and only the block as a random effect when analysing individual treatments.

## Results

### Effect of different starting mite densities on the trade-off between offspring size and number

**Fig 2:**
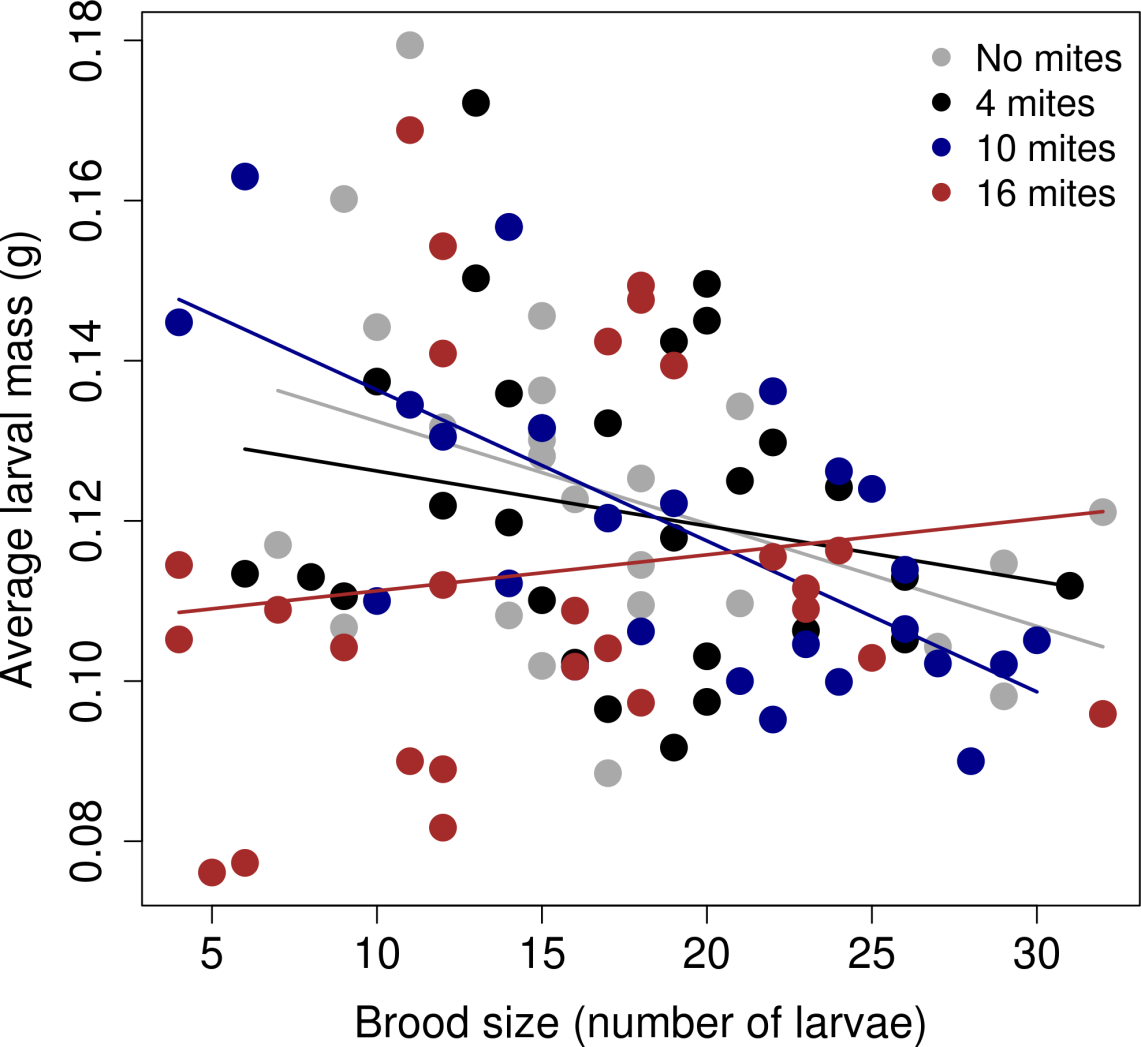
The relationship between the average larvae mass (g) and the total brood size (number of larvae was for each mite treatment). The graph shows the linear regression between the raw values, separated by the mite treatment.

The trade-off between brood size and average larval mass differed among the different mite density treatments (Table 1; Fig 2). The results from the models ran for each 'mite' treatment showed that there was a strong, negative relationship between the final number of larvae and the average larval mass when there were no mites present (Estimate = -0.01; SE = 0.0006; d.f. = 21; t value = -2.64; *p* = 0.01), when there was an initial number of four mites present in the reproductive event (Estimate = -0.01; SE = 0.0007; d.f. = 24.93; t value = -2.63; *p* = 0.01), and when there was an initial number of ten mites (Estimate = -0.01; SE = 0.0004; d.f. = 24.24; t value = -4.64; *p* < 0.00001). However, when there was an initial number of sixteen mites, the relationship became flat (Estimate = -0.0004; SE = 0.0006; d.f. = 23.58; t value = -0.66; *p =* 0.5). The slope of the relationship between the size of the brood and the average larval mass was significantly different between the 'no mites' treatment and the '16 mites' treatment (Table 2).

**Table 1:** Results from the final models for each variable analysed using the 'Anova' function. *n* = 24 for the 'without mites' treatment, *n* = 28 for the 'four mites' treatment, *n* = 28 for the 'ten mites' treatment, *n* = 27 for the 'sixteen mites' treatment. Final models are shown.

Dependent Variable	Explanatory variables	*Χ*^*2*^	d.f.	*p* value
Clutch size	Carcass mass after preparation	0.91	1	0.33
	Male size	0.51	1	0.47
	Female size	7.66	1	0.005*
Brood size	Treatment	5.26	3	0.15
	Carcass mass after preparation	15.17	1	< 0.0001*
Brood mass	Treatment	7.50	3	0.057
	Carcass mass after preparation	19.82	1	<0.00001*
Average larval mass	Treatment	13.97	3	0.002*
	Carcass mass after preparation	4.68	1	0.03*
	Brood size	9.54	1	0.002*
	Brood size*Treatment	8.63	3	0.03*
Number of dead larvae	Treatment	9.51	5	0.02*

*p < 0.05

**Table 2:** Results from the final models for each variable analysed using the 'summary' function. *n* = 24 for the 'without mites' treatment, *n* = 28 for the 'four mites' treatment, *n* = 28 for the 'ten mites' treatment, *n* = 27 for the 'sixteen mites' treatment. Final models are shown.

Dependent variable	Explanatory variables	Estimate	SE	d.f.	t value	*p* value
Average larval mass	Brood size	-0.001	0.0005	95.34	-3.08	0.002*
	4 mites treatment	-0.007	0.015	91.09	-0.51	0.60
	10 mites treatment	0.005	0.014	86.55	0.35	0.72
	16 mites treatment	-0.03	0.013	95.83	-2.86	0.005*
	Carcass mass after preparation	0.005	0.002	97.03	2.16	0.03*
	4 mites treatment*Brood size	0.0003	0.0008	92.21	0.47	0.63
	10 mites treatment*Brood size	-0.0003	0.0007	87.31	-0.40	0.68
	16 mites treatment*Brood size	0.001	0.0007	96.94	2.19	0.03*

*p < 0.05

**Fig 3:**
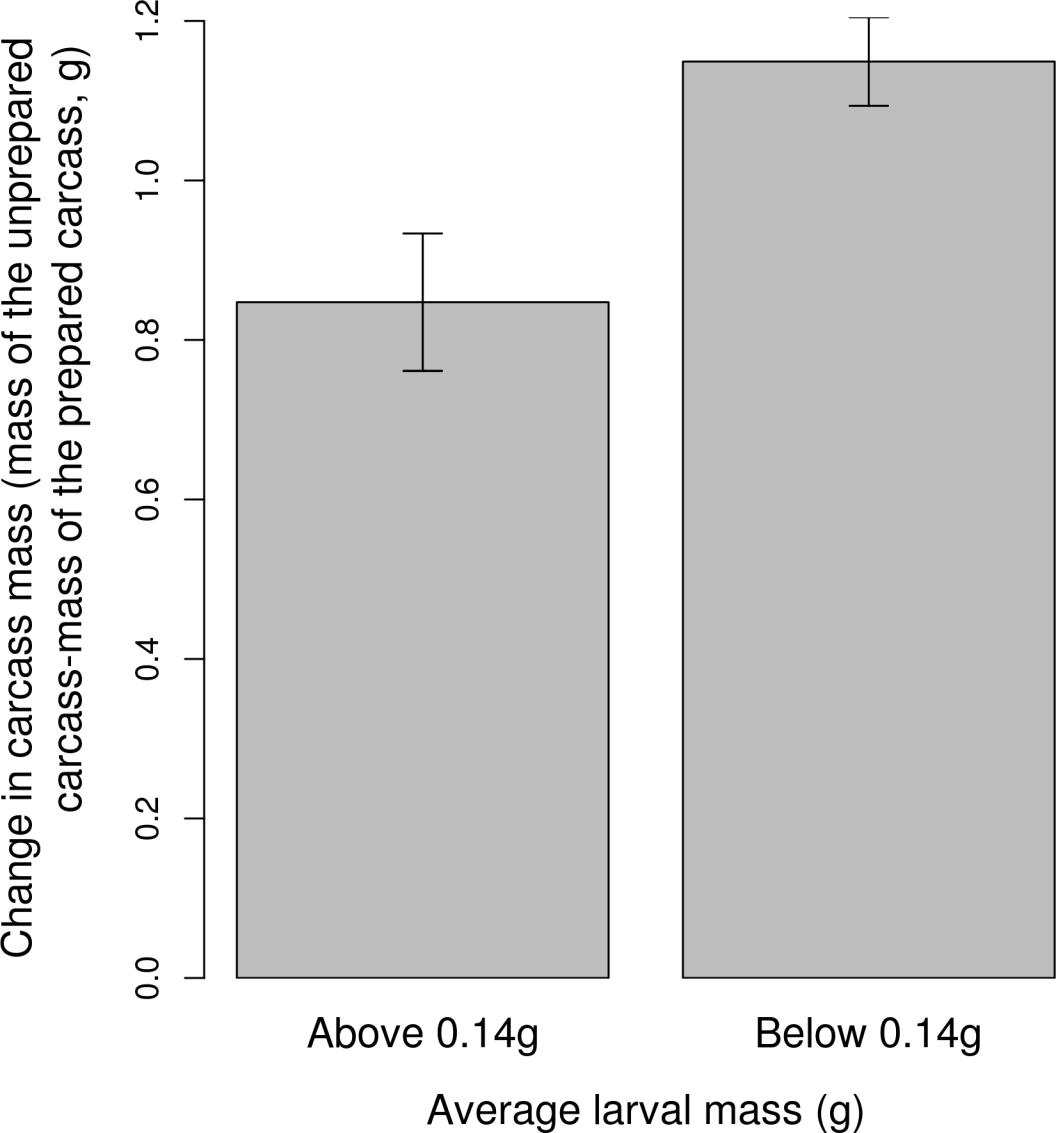
The relationship between the change in carcass mass after its preparation and larval mass at dispersal. Only broods from the '16 mite' treatment, and with fewer than 20 larvae per brood are presented. Means ± SE are raw values.

Because this effect seemed to be driven by some broods in the 16 mites treatment that had fewer than 20 larvae, each with an average mass of less than 0.14g, we examined in greater detail the subset of broods in this treatment that had fewer than 20 larvae. We split this subset into two further groups: those with an average larval mass of less than 0.14g and those that with more than 0.14g. For these two groups we compared a) the mass of the carcass after preparation, b) the change in carcass mass before and after preparation (calculated as mass of the unprepared carcass–mass of the prepared carcass), c) the size of the father, d) the size of the mother, and e) the final number of progeny mites dispersing on the beetles (log transformed). The only difference that we found between these two groups was the change in mass between prepared and unprepared carcasses (Fig 3). For broods with larvae lighter than 0.14g (i.e. those that did not follow the normal trade-off), the decrease in carcass mass during preparation was greater than that seen at pairs that followed the 'usual' trade-off. We did not find any other significant differences between these two groups of beetles (*p >* 0.09).

### Effect of different starting mite densities on the success of the brood

**Fig 4:**
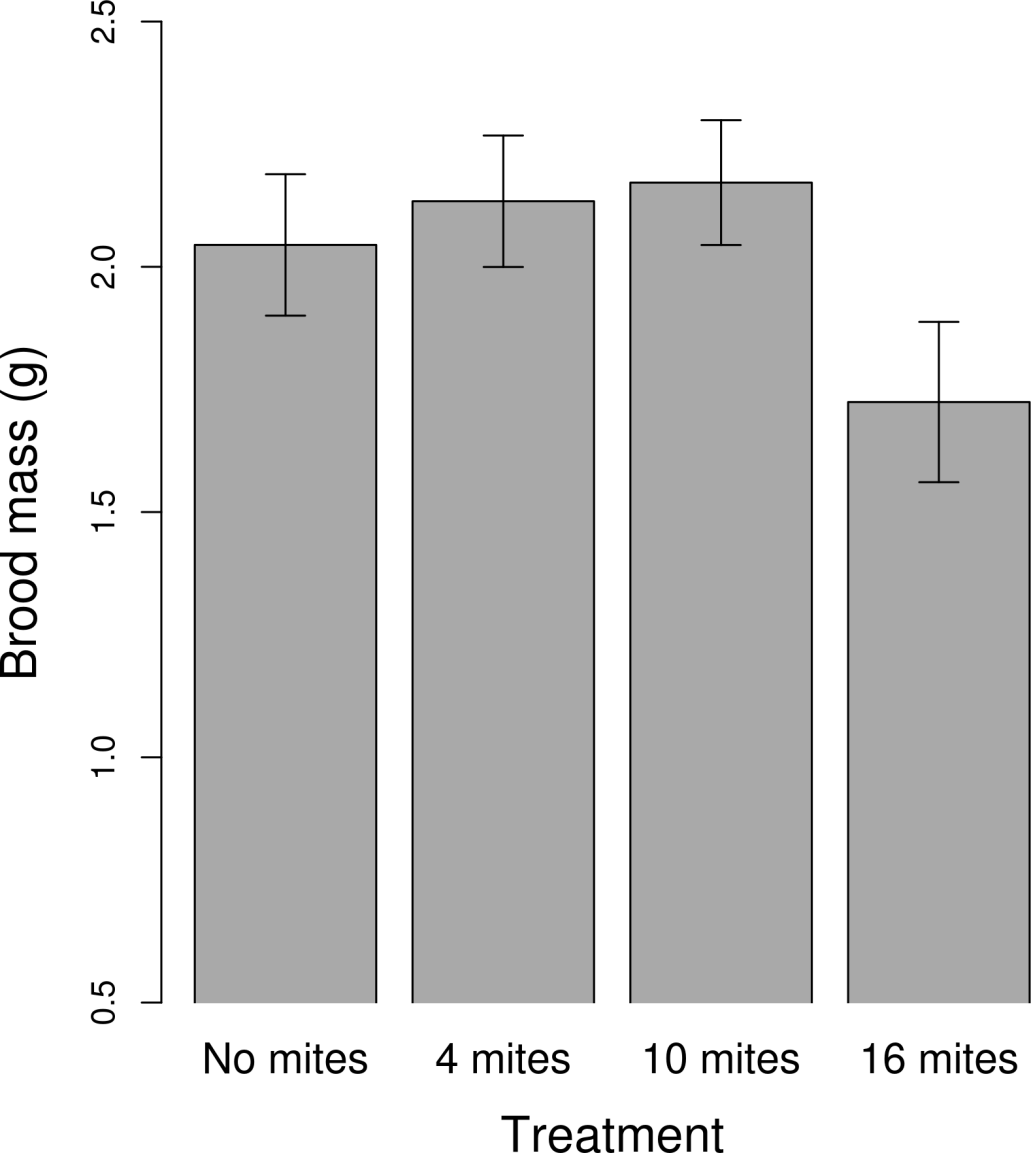
The relationship between brood mass and the different mite density treatment. Means ± SE are raw values.

**Fig 5:**
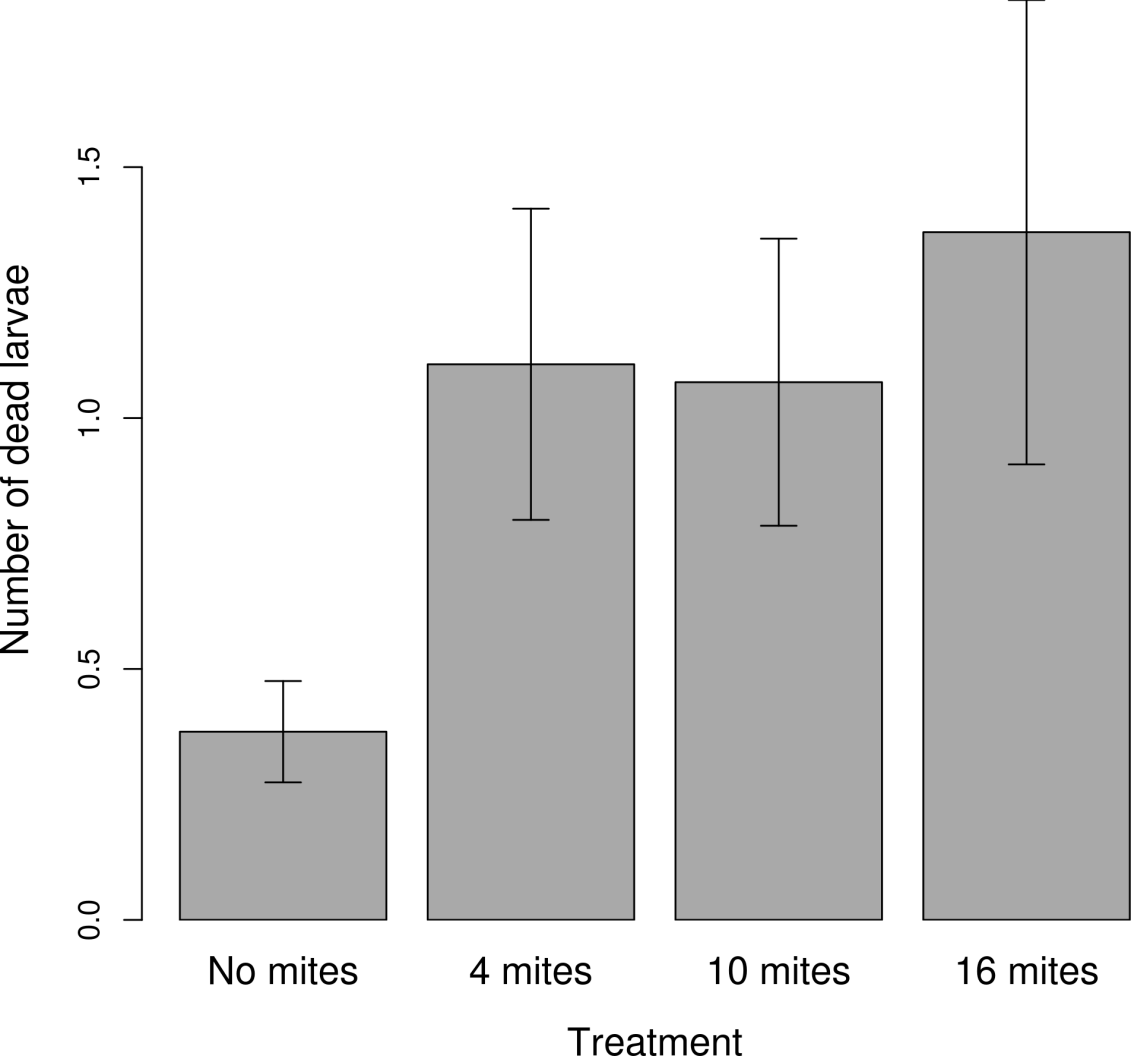
The relationship between the treatment and the number of dead larvae. The graph shows the mean ± SE from the raw values.

The mite density treatment had no effect on either the size of the clutch or the size of the brood (Table 1). Female size positively predicted clutch size (Table 1). The mite density treatment had a marginally significant effect on the final mass of the brood (Table 1, Fig 4), driven mainly by a reduction in brood mass at the highest initial mite density. The frequency of dead larvae was significantly affected by the treatment (Table 1). Using the 'summary' function we found that there were significantly fewer dead larvae in the control than in the 4 mite treatment (Estimate = 1.12; SE = 0.37; z value = 2.97; *p =* 0.002; Fig 5), and the 10 mite (Estimate = 0.97; SE = 0.37; z value = 2.59; *p =* 0.009), and the 16 mite (Estimate = 1.06; SE = 0.37; z value = 2.81; *p =* 0.004). There were no other significant differences among the treatments.

**Fig 6:**
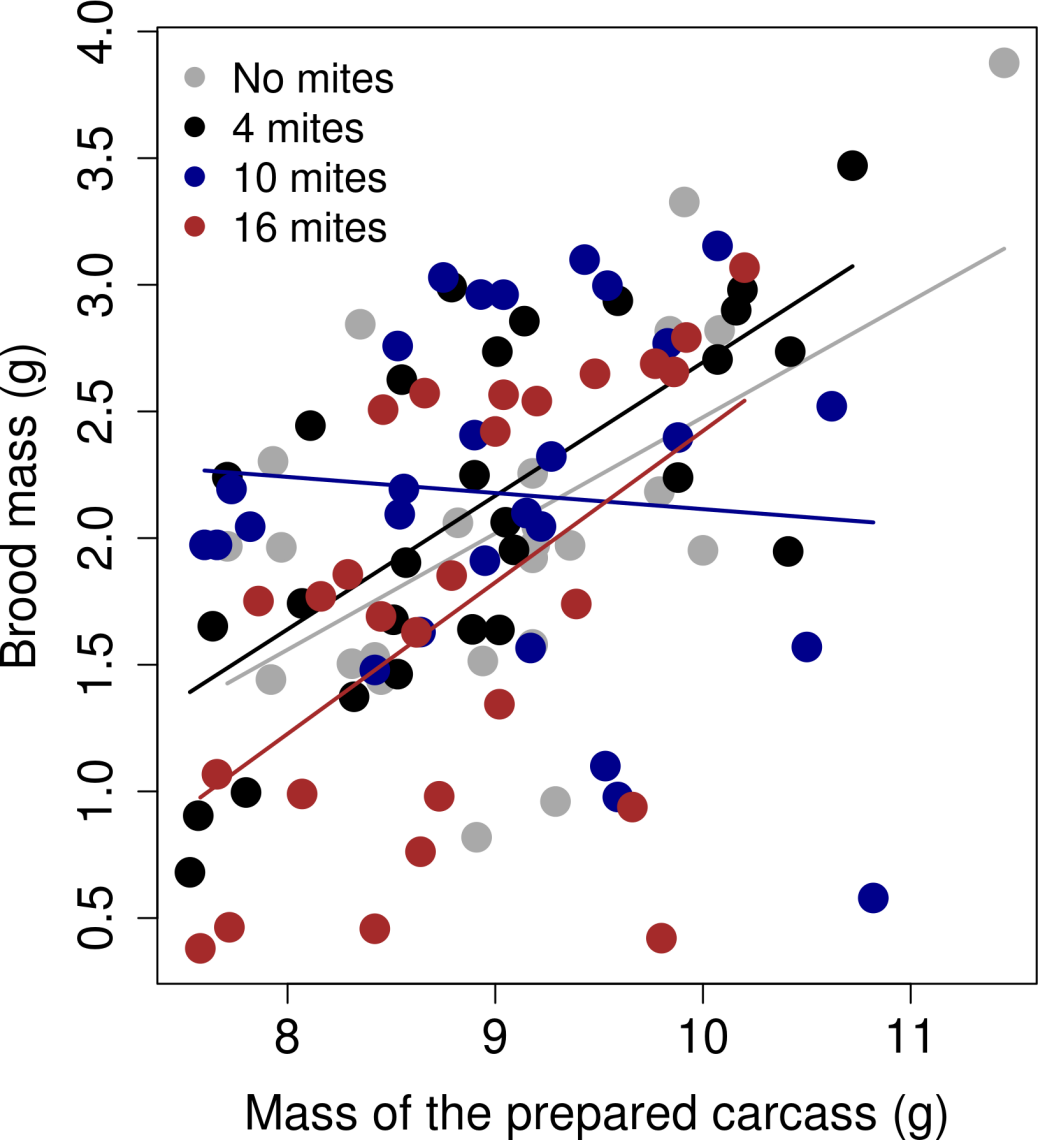
The relationship between the brood mass (g) and the mass of the prepared carcass (g). The graph shows the linear regression between the raw values, separated by the mite treatment.

**Fig 7:**
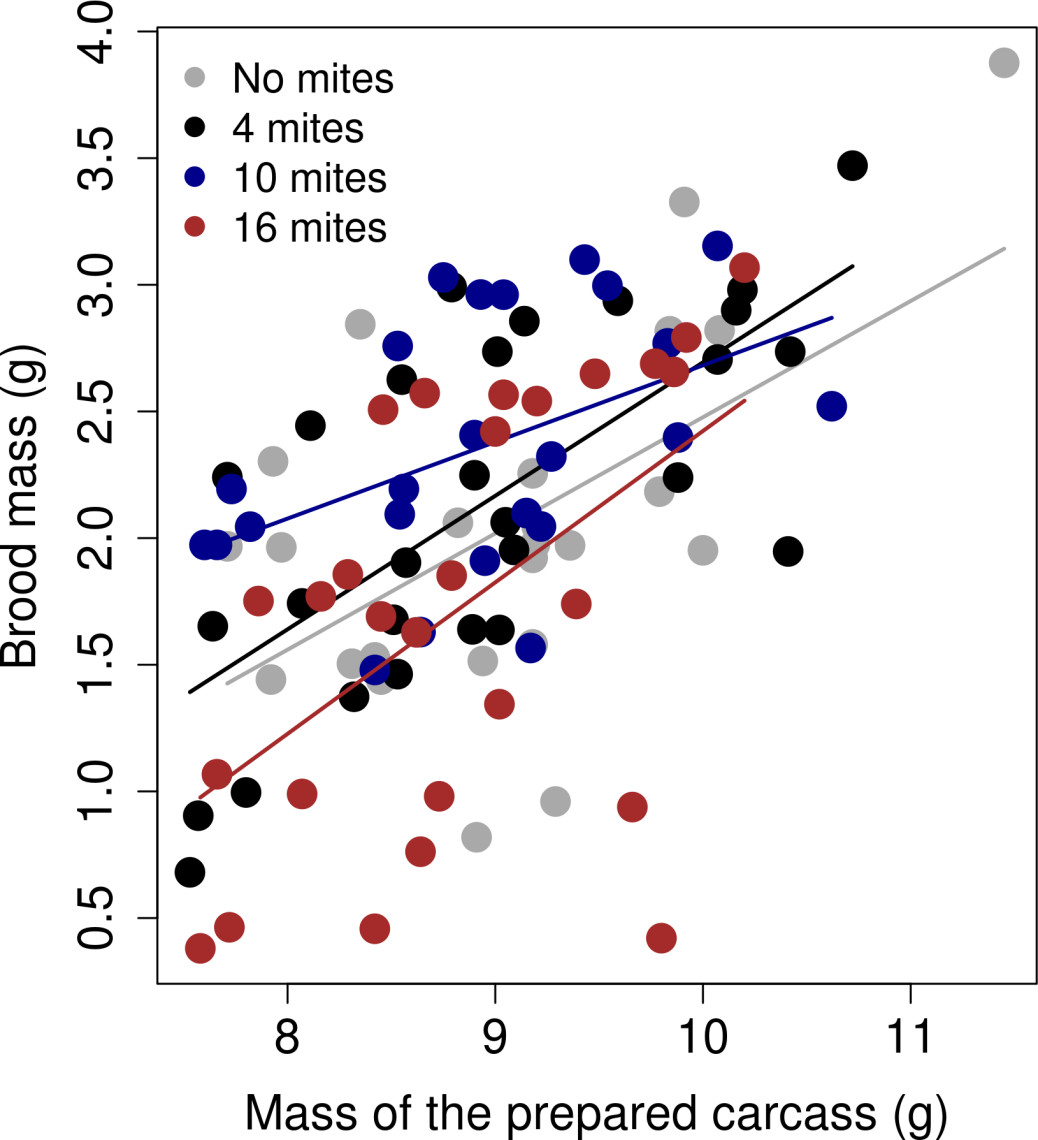
The relationship between the brood mass (g) and the mass of the prepared carcass (g), after removing four extreme values in the '10 mite' treatment. The graph shows the linear regression between the raw values, separated by the mite treatment.

We found a significant effect of the interaction between the carcass mass after preparation and the different mite treatments on the final mass of the brood (χ^2^_3_ = 9.76; *p* = 0.02). The slope of the 10 mite treatment was significantly different from the slope of the 'no mite' treatment (Estimate = -0.45; SE = 0.19; z value = -2.35; *p =* 0.02; Fig 6). Further inspection of the data suggested that this effect was driven by a few points. After removing these four points, this effect disappeared (general treatment effect: χ^2^_3_ = 1.35; *p* = 0.71; difference between the '10 mite' and 'no mite' treatments: Estimate = -0.14; SE = 0.19; z value = -0.7; *p =* 0.46; Fig 7). There was no other significant effect of the interaction between the treatment and the carcass mass on either brood size or average larval mass.

### Effect of the final mite density on the success of the brood

The final number of mites did not influence either the final size of the brood, the clutch size, brood mass or average larval mass (*p* > 0.09 in all cases). When we analysed each mite treatment separately, we found that the final number of progeny mites (log transformed) negatively predicted the final mass of the brood, but only in the '10 mite' treatment (Estimate = -0.56; SE = 0.24; z value = -2.28; *p =* 0.03). We also found a marginally significant positive effect of the final number of progeny mites (log transformed) on the average larval mass in the '16 mite' treatment (Estimate = -0.02; SE = 0.01; z value = 1.93; *p =* 0.06). The final mite density did not influence the final size of the brood nor the clutch size, for any of the mite treatments.

## Discussion

Our experiments on the burying beetle show that interactions with its phoretic mite can change the trade-off between brood size and offspring size. Specifically, when there were high mite densities at the start of reproduction, the gradient of this relationship flattened considerably, and at a relatively low intercept. How did this pattern emerge? Visual inspection of the data suggests that high densities of mites were especially detrimental at small brood sizes, although we cannot identify precisely why this was (Fig 2). One possibility is that parents with small broods were simply overwhelmed by larger numbers of rival mites for resources, whereas those with larger broods could more effectively monopolise access to the carcass [40]. Alternatively perhaps parents strategically under-invested in small broods when the carcass was heavily infested with mites and thus of poor quality, in much the same way as parents withhold investment from current reproduction to prioritise investment in future reproduction when given a very small brood to rear [41], or when given a low-quality carcass already colonised by microbial competitors [42].

Detailed analysis of the data collected from the ‘16-mite’ treatment suggests that the change in the slope of the trade-off was caused by a greater depletion of resources from the carcass during its preparation, which effectively took resources away from the larvae (Fig 3). However, we cannot tell whether mites depleted these resources directly (and some parents were more effective at preventing this) or whether mites caused parents to consume more of the carcass themselves (and some parents ate more carrion than others) [43]. Independently of whether mites took these resources away or parents consumed more of the carcass themselves, perhaps this effect would disappear if parents had more resources. Future studies could analyse how the quality of the carcass affects this trade-off.

We found no evidence to suggest that the mite treatment influenced either clutch size or the size of the brood at dispersal. This suggests that parents were not withholding investment by adjusting the number of eggs they laid or via an increased incidence of filial cannibalism [27], only that their larvae were under-provisioned. Curiously, though, we did find significantly more dead larvae when mites bred alongside beetles than when they were absent. This is consistent with our previous observations of mites attacking and killing newly hatched larvae [25]. It is also possible that mites were indirectly responsible for the death of these larvae by somehow inducing greater levels of infanticide by parents. How can we reconcile these apparently conflicting findings, in which there is no difference in the number of eggs laid among treatments, greater mortality in the treatments with mites, and yet no difference among treatments in the number of larvae that disperse at the end of reproduction? Part of the reason may be due to the fact that the frequency of dead larvae was very small, even in the high mite density treatments (we usually never found more than two dead larvae per family). So, although we consistently found more dead larvae in families that bred alongside mites, this number was not large enough to produce an effect on the final size of the brood. Another possibility could be that there is a high level of error in our estimates of clutch size. It may be that beetles in fact laid more eggs when breeding alongside mites than when they were not, in strategic anticipation of their offspring being killed by mites [44]. This explanation seems unlikely, though, because it is hard to explain why we might have systematically under-estimated clutch size only in the treatments with mites. A different possibility is that the mites killed (directly or indirectly) the fraction of larvae that would have otherwise been eliminated by their parents through partial filial cannibalism. These possibilities remain to be investigated in future work.

In previous work we found a strong relationship between the final number of mites and the success of the brood: brood size fell with increasingly large numbers of progeny mites, but those individual larvae that survived attained a greater mass by the time they dispersed away to pupate [25]. Why did we not find the same effect in this study? One possible explanation is that in this experiment, and for reasons we cannot explain, the final number of mites (in the 10 and 16 mite treatments) was significantly lower than in our previous experiment [25] (*t* = 2.34; d.f. = 40.92; *p* = 0.02). Perhaps only when the final number of mites is very large, as in [25], is there a strong relationship between brood size and final number of mites. In our previous study we also found evidence of mites attacking burying beetle larvae directly [25]. Together, the two studies suggest that the resources available for nourishing individual larvae are affected in two distinct ways by the presence of mites. At intermediate mite densities, mites do not change the number of beetle larvae (directly or indirectly via the parents), but they do change the extent to which each individual larva is nourished (whether directly or indirectly via the parents we do not yet know). From an individual offspring’s perspective, it can never attain a high mass at dispersal under these conditions (comparable to the scenario illustrated in Fig 1D). At higher mite densities still, however, mites start to attack larvae directly and so reduce brood size. The remaining resources on the carcass are then divided among fewer larvae so that each of the survivors is better-nourished and attains a greater mass by the time the larval stage ends. This outcome is better from the perspective of a surviving larva than might be achieved at lower mite densities (and corresponds with the scenario depicted in Fig 1C).

At this point we should acknowledge a potential artefact in our experimental designs (here and in [25]), which might influence our interpretation of the results. In these experiments, we prevented males from dispersing away from the brood, whereas in nature males commonly leave before larval development is complete [24]. When males leave, they take with them a significant number of progeny mites, and males leave sooner when mites are present during reproduction than when they are absent [26]. Therefore it could be argued that by forcing fathers to stay with the brood in this experiment we created artificially high densities of mites on the carcass at the end of the breeding event, which exceed those ever seen in nature. In other words, the effects of mites we have reported on larval mass can be created in laboratory experiments but might never be exposed to selection under natural conditions. Therefore in future work it will be important to test whether the effects we found here and in [25] remain when males are allowed to leave at a time of their own choosing.

Previous work has considered how optimal levels of parental investment might evolve in response to a persistent change in ecological conditions [1], causing a consequent evolved change in optimal levels of investment from the offspring’s perspective [e.g. 10]. Our analyses of the interactions between mites and burying beetles differ from this approach by considering how temporary and stochastic variation in ecological conditions can suddenly and temporarily hand one party the victory in an evolutionary conflict of interest. Our previous work suggests that phoretic mites potentially change the outcome of evolutionary conflicts within the family in this way, and in particular play a key role in determining which sex ‘wins’ diverse forms of sexual conflict that arise during burying beetle reproduction [25,26,45]. Here we have taken a first step towards investigating whether mites could similarly influence the outcome of parent-offspring conflict, via the hypothetical mechanisms illustrated in Fig 1. Our experiments demonstrate that at high densities, mites can cause the trade-off between brood size and offspring size depicted by the black line in Fig 1D to temporarily resemble the relationship depicted with the red line. However, whether the parent and offspring optima for burying beetles resemble those illustrated in Fig 1D remains to be determined. Until these experiments are carried out, it is too soon to conclude that mites can temporarily prevent larvae from ‘winning’ any parent-offspring conflict over offspring size. Nevertheless, the concepts we introduce here show in principle how the outcome of parent-offspring conflict can fluctuate on an ever-changing ecological stage, in an empirically testable way, and that the outcome need not be evolutionarily stable in the short-term [46].

## Supporting Information

S1 Table Excel document containing the data from this experiment. (XLSX)

## References

[1] Stearns SC. Life-history tactics: A review of the ideas. Q Rev Biol. 1976;51: 3–47. 778893 10.1086/409052

[2] Smith HG, Kallander H, Nilsson J-A. The trade-off between offspring number and quality in the great tit *Parus major*. J Anim Ecol. 1989;58: 383–401. 10.2307/4837

[3] Fleming IA, Gross MR. Latitudinal clines: a trade-off between egg number and size in Pacific salmon. Ecology. 1990;71: 2–11. 10.2307/1940241

[4] Ebert D. The trade-off between offspring size and number in *Daphnia magna*: the influence of genetic, environmental and maternal effects. Arch Hydrobiol Suppl. 1993;90: 453–473.

[5] Einum S, Fleming IA. Highly fecund mothers sacrifice offspring survival to maximize fitness. Nature. 2000;405: 565–567. 10.1038/35014600 10850714

[6] Wilson AJ, Coltman DW, Pemberton JM, Overall ADJ, Byrne KA, Kruuk LEB. Maternal genetic effects set the potential for evolution in a free-living vertebrate population. J Evol Biol. 2005;18: 405–414. 10.1111/j.1420-9101.2004.00824.x 15715846

[7] Janzen FJ, Warner DA. Parent–offspring conflict and selection on egg size in turtles. J Evol Biol. 2009;22: 2222–2230. 10.1111/j.1420-9101.2009.01838.x 19796084

[8] Kilner RM, Hinde CA. Chapter 7 Parent–offspring conflict In: Royle NJ, Smiseth P, Kölliker M, editors. The Evolution of Parental Care. Oxford (UK): Oxford University Press 2012; 119–132.

[9] Kilner RM, Hinde CA. Chapter 6 Information warfare and parent–offspring conflict In: Brockmann HJ, Roper TJ, Naguib M, Wynne-Edwards KE, Barnard C, et al, Advances in the Study of Behavior. San Diego: Elsevier Academic Press Inc. 2012; 38:283–336.

[10] Parker GA, Begon M. Optimal egg size and clutch size: effects of environment and maternal phenotype. Am Nat. 1986;128: 573–592.

[11] Mock DW, Forbes LS. Parent-offspring conflict: a case of arrested development. Trends Ecol Evol. 1992;7: 409–413. 10.1016/0169-5347(92)90022-4 21236082

[12] Wilson AJ, Pemberton JM, Pilkington JG, Clutton-Brock TH, Kruuk LEB. Trading offspring size for number in a variable environment: selection on reproductive investment in female Soay sheep. J Anim Ecol. 2009;78: 354–364. 10.1111/j.1365-2656.2008.01489.x 19302125

[13] Gliwicz ZM, Guisande C. Family planning in *Daphnia*: resistance to starvation in offspring born to mothers grown at different food levels. Oecologia. 1992;91: 463–467. 10.1007/BF00650317 28313496

[14] Pukowski E. Ökologische untersuchungen an *necrophorus* f. Z Für Morphol Ökol Tiere. 1933;27: 518–586. 10.1007/BF00403155

[15] Scott MP. The ecology and behavior of burying beetles. Annu Rev Entomol. 1998;43: 595–618. 10.1146/annurev.ento.43.1.595 15012399

[16] Eggert A-K, Müller JK. Joint breeding in female burying beetles. Behav Ecol Sociobiol. 1992;31: 237–242. 10.1007/BF00171678

[17] Kilner RM, Boncoraglio G, Henshaw JM, Jarrett BJ, De Gasperin O, Attisano A, et al Parental effects alter the adaptive value of an adult behavioural trait. eLife. 2015;4: e07340 10.7554/eLife.07340 26393686 PMC4613925

[18] House CM, Hunt J, Moore AJ. Sperm competition, alternative mating tactics and context-dependent fertilization success in the burying beetle, *Nicrophorus vespilloides*. Proc R Soc Lond B Biol Sci. 2007;274: 1309–1315. 10.1098/rspb.2007.0054 PMC2176180 17360284

[19] Müller JK, Braunisch V, Hwang W, Eggert A-K. Alternative tactics and individual reproductive success in natural associations of the burying beetle, *Nicrophorus vespilloides*. Behav Ecol. 2007;18: 196–203. 10.1093/beheco/arl073

[20] Eggert A-K. Alternative male mate-finding tactics in burying beetles. Behav Ecol. 1992;3: 243–254. 10.1093/beheco/3.3.243

[21] Hopwood PE, Moore AJ, Tregenza T, Royle NJ. Niche variation and the maintenance of variation in body size in a burying beetle. Ecol Entomol. 2015; n/a–n/a. 10.1111/een.12275

[22] Springett BP. Aspects of the relationship between burying beetles, *Necrophorus* spp. and the mite, *Poecilochirus necrophori* Vitz. J Anim Ecol. 1968;37: 417–424. 10.2307/2957

[23] Wilson DS, Knollenberg WG. Adaptive indirect effects: the fitness of burying beetles with and without their phoretic mites. Evol Ecol. 1987;1: 139–159. 10.1007/BF02067397

[24] Schwarz HH, Müller JK. The dispersal behaviour of the phoretic mite *Poecilochirus carabi* (Mesostigmata, Parasitidae): adaptation to the breeding biology of its carrier *Necrophorus vespilloides* (Coleoptera, Silphidae). Oecologia. 1992;89: 487–493. 10.1007/BF00317154 28311878

[25] De Gasperin O, Kilner RM. Friend or foe: inter-specific interactions and conflicts of interest within the family. Ecol Entomol. 2015;40: 787–795. 10.1111/een.12259 26681822 PMC4678582

[26] De Gasperin O, Duarte A, Kilner RM. Interspecific interactions explain variation in the duration of paternal care in the burying beetle. Anim Behav. 2015;109: 199–207. 10.1016/j.anbehav.2015.08.014 26778845 PMC4686539

[27] Bartlett J. Filial cannibalism in burying beetles. Behav Ecol Sociobiol. 1987;21: 179–183.

[28] Trumbo ST. Regulation of brood size in a burying beetle, *Nicrophorus tomentosus* (Silphidae). J Insect Behav. 1990;3: 491–500. 10.1007/BF01052013

[29] Rauter CM. Influence of population density on offspring number and size in burying beetles. Bioscene J Coll Biol Teach. 2010;36: 6–11.

[30] Müller JK, Schwarz HH, others. Differences in carrier-preference and evidence of reproductive isolation between mites of *Poecilochirus carabi* (Acari, Parasitidae) living phoretically on two sympatric *Necrophorus species* (Coleoptera, Silphidae). Zool Jahrb Abt Für Syst Ökol Geogr Tiere. 1990;117: 23–30.

[31] Brown JM, Wilson DS. Local specialization of phoretic mites on sympatric carrion beetle hosts. Ecology. 1992;73: 463–478. 10.2307/1940753

[32] Schwarz HH, Starrach M, Koulianos S. Host specificity and permanence of associations between mesostigmatic mites (Acari: Anactinotrichida) and burying beetles (Coleoptera: Silphidae: *Nicrophorus*). J Nat Hist. 1998;32: 159–172. 10.1080/00222939800770101

[33] Guisande C, Sanchez J, Maneiro I, Miranda A. Trade-off between offspring number and offspring size in the marine copepod *Euterpina acutifrons* at different food concentrations. Mar Ecol Prog Ser Oldendorf. 1996;143: 37–44.

[34] Müller JK, Eggert A-K, Elsner T. Nestmate recognition in burying beetles: the “breeder’s badge” as a cue used by females to distinguish their mates from male intruders. Behav Ecol. 2003;14: 212–220. 10.1093/beheco/14.2.212

[35] Smiseth PT, Dawson C, Varley E, Moore AJ. How do caring parents respond to mate loss? Differential response by males and females. Anim Behav. 2005;69: 551–559. 10.1016/j.anbehav.2004.06.004

[36] R Development Core Team R. A language and environment for statistical computing R Foundation for Statistical Computing, Vienna, Austria 2011; http://www.R-project.org/

[37] Bates D, Maechler M, Bolker B. lme4: Linear mixed-effects models using S4 classes. 2012; http://CRAN.R-project.org/package=lme4

[38] Akaike H. A new look at the statistical model identification. IEEE Trans Autom Control. 1974;19: 716–723. 10.1109/TAC.1974.1100705

[39] Fox J, Weisberg S, Adler D, Bates D, Baud-Bovy G, Ellison S, et al. car: Companion to applied regression. 2015; https://cran.r-project.org/web/packages/car/index.html

[40] Schrader M, Jarrett BJM, Kilner RM. Parental care masks a density-dependent shift from cooperation to competition among burying beetle larvae. Evolution. 2015; 69:1077–1084. 10.1111/evo.12615 25648525 PMC4476075

[41] Ward RJ, Cotter SC, Kilner RM. Current brood size and residual reproductive value predict offspring desertion in the burying beetle *Nicrophorus vespilloides*. Behav Ecol. 2009; arp132.

[42] Rozen DE, Engelmoer DJP, Smiseth PT. Antimicrobial strategies in burying beetles breeding on carrion. Proc Natl Acad Sci. 2008;105: 17890–17895. 10.1073/pnas.0805403105 19001269 PMC2584725

[43] Boncoraglio G, Kilner RM. Female burying beetles benefit from male desertion: sexual conflict and counter-adaptation over parental investment. PloS One. 2012;7: e31713 10.1371/journal.pone.0031713 22355390 PMC3280230

[44] Lyon BE. Optimal clutch size and conspecific brood parasitism. Nature. 1998;392: 380–383. 10.1038/32878

[45] De Gasperin O, Kilner RM. Interspecific interactions change the outcome of sexual conflict over pre-hatching parental investment in the burying beetle *Nicrophorus vespilloides*. Ecol Evol. 2015; n/a–n/a. 10.1002/ece3.1795 PMC4813101 27069605

[46] Godfray HCJ. Signaling of need between parents and young: parent-offspring conflict and sibling rivalry. Am Nat. 1995; 1–24.

